# Effects of Sterilization Processes with Hydrogen Peroxide and Ethylene Oxide on Commercial 3D-Printed PLA, PLA-FC, and PETG by Fused Deposition Modeling

**DOI:** 10.3390/polym17212864

**Published:** 2025-10-27

**Authors:** Jorge Mauricio Fuentes, Homero Cadena, Abel Remache, Omar Flor-Unda, Santiago Sarria, Jonathan Delgado, Pablo Bonilla, Santiago Ferrándiz

**Affiliations:** 1 Ingeniería en Diseño Industrial e Ingeniería Mecánica, Facultad de Ingeniería y Ciencias Aplicadas, Universidad Central del Ecuador, Quito 170521, Ecuador; jmfuentes@uce.edu.ec (J.M.F.); hdcadena@uce.edu.ec (H.C.); apremache@uce.edu.ec (A.R.); gssarria@uce.edu.ec (S.S.); jadelgadog@uce.edu.ec (J.D.); pmbonilla@uce.edu.ec (P.B.); 2 Ingeniería Industrial, Facultad de Ingeniería y Ciencias Aplicadas, Universidad de Las Américas, Quito 170125, Ecuador; 3 Departamento de Ingeniería Mecánica y de Materiales, Instituto de Tecnología de Materiales, Universitat Politécnica de València, Plaza Ferrándiz y Carbonell s/n, 03801 Alcoi, Spain; sferrand@mcm.upv.es

**Keywords:** polymer sterilization, hydrogen peroxide, ethylene oxide, 3D printing, PLA, PLAFC, PETG, 3D FDM

## Abstract

Polymers such as PLA, PLA reinforced with carbon fiber (PLA + CF), and PETG are widely employed in utensils, structural components, and biomedical device housings where load-bearing capability and chemical resistance are desirable. This is particularly relevant for reusable applications in which sterilization with hydrogen peroxide (HP) or ethylene oxide (EO) is often required. In this study, the impact of HP and EO sterilization processes on the mechanical, thermal, and structural properties of PLA, PLA + CF, and PETG was evaluated. The mechanical properties assessed included elongation at break, elastic modulus, and tensile strength after sterilization. The thermal properties examined comprised thermal stability and the coefficient of thermal expansion (CTE). Additionally, Fourier Transform Infrared Spectroscopy (FTIR) was performed to detect potential alterations in functional groups. For PLA, sterilization with HP and EO resulted in a 22% increase in ultimate tensile strength (UTS) and a 21% increase in elastic modulus, accompanied by a noticeable reduction in ductility and the appearance of more brittle fracture surfaces. PLA + CF exhibited greater stability under both sterilization methods due to the reinforcing effect of carbon fibers. In the case of PETG, tensile strength and stiffness remained stable; however, HP sterilization led to a remarkable increase in elongation at break (294%), whereas EO sterilization reduced it. Regarding thermal properties, glass transition temperature (Tg) showed variations: PLA presented either an increase or decrease in Tg depending on the sterilization treatment, PLA + CF displayed a Tg reduction after EO sterilization, while PETG exhibited a moderate Tg increase under HP sterilization. CTE decreased at lower temperatures but increased after EO treatment. FTIR analysis revealed only minor chemical modifications induced by sterilization. Overall, HP and EO sterilization can be safely applied to additively manufactured medical components based on these polymers, provided that the structures are not subjected to high mechanical loads and do not require strict dimensional tolerances.

## 1. Introduction

Sterilization of medical devices and equipment is an essential process to prevent infections and ensure patient safety. Among the most commonly used methods are ethylene oxide (C_2_H_4_O) and hydrogen peroxide in the vapor/plasma phase (H_2_O_2_/HPGP), recognized for their effectiveness and compatibility with a wide range of materials. These low-temperature techniques are particularly relevant for parts produced by fused deposition modeling (FDM) using polymers such as PLA, PLA reinforced with carbon fiber (PLA + CF), and PETG, as they minimize thermal deformation and preserve mechanical integrity—unlike autoclaving, which involves exposure to 121–134 °C and can distort thermoplastic components [1].

Several clinical reports and surgical guidelines have shown that H_2_O_2_ maintains dimensional errors in the submillimeter range, while EO ensures complete sterility with minimal changes; in contrast, autoclaving produces significant deformations, which forces the sterilization method to be chosen according to the tolerance and geometric complexity of the device [2]. For PLA + CF composites, the addition of carbon fiber increases rigidity and structural stability, although the same criteria of using low-temperature techniques and verifying, before clinical use, dimensional accuracy and mechanical performance after sterilization are maintained [3,4,5].

Sterilization processes with hydrogen peroxide (H_2_O_2_) and ethylene oxide (EO) have proven to be effective for 3D-printed materials such as PLA, PLA-FC, and PETG, with variations depending on the polymer and method employed. In general, H_2_O_2_ is favorable for PLA by preserving its mechanical and physical properties without significant alterations, and is also suitable for heat-sensitive materials [6]; similarly, in PLA-FC and PETG it causes minimal changes in morphology and dimensions, which allows the integrity of the parts to be maintained [5]. On the other hand, EO is also effective for PLA with minimal modifications in its properties, although it requires longer process times [7,8]; likewise, in PLA-FC and PETG it guarantees the conservation of its mechanical characteristics and avoids relevant deformations, being especially advantageous for complex geometries that demand gas penetration [5]. Table 1 summarizes the main findings of previous studies addressing these sterilization methods and materials.

**Table 1:** Synthesis of sterilization findings in PLA, PLA + FC, and PETG.

Material	Hydrogen Peroxide (H_2_O_2_)	Ethylene Oxide (EO)	Ref
PLA	Preserves characteristics, minimal alterations.	Minimal changes, effective for complex geometries.	[6,8,9]
PLA-FC	Minimal changes, maintains integrity.	Minimal deformation, preserves properties.	[5]
PETG	Minimal changes, maintains integrity.	Minimal deformation, preserves properties	[5]

In the manufacture of medical devices using FDM, PLA, PLA-FC and PETG stand out for their biocompatibility, processability and reproducibility, which has favored their use in surgical guides, anatomical models and even functional components [10]. However, their final performance is highly dependent on compatibility with sterilization processes. In this regard, the use of ethylene oxide has been recommended for medical devices 3D printed with polymers such as polylactic-co-glycolic acid (PLGA) [11]. Although this method may compromise molecular integrity or surface properties [11,12], dimensional changes after sterilization have been found to remain within very small margins (≤0.05 mm) [13].

The scientific literature shows divergent results on the impact of sterilization on mechanical properties. Some studies report structural or aesthetic alterations in PLA and PETG [7], deformations in parts smaller than 5 mm exposed to autoclave [3], changes in PLA, PETG and PP aortic stents subjected to 121 °C [5] and loss of dimensional accuracy after steam sterilization [4]. In contrast, other research notes that sterilization does not affect key parameters such as stiffness, tensile strength, or flexural strength [6,14]. This disparity highlights the need for studies that are not limited to a specific product type, but that comprehensively analyze various mechanical properties and consider both the composition of the polymer and the geometric complexity and printing parameters.

In this context, the present study focuses on evaluating the effects of ethylene oxide and hydrogen peroxide on the mechanical properties of 3D-printed PLA, PLA-FC and PETG. The objective is to provide solid evidence on how these sterilization processes affect the characterization of materials intended for medical applications, contributing to the safety, efficacy and optimization of devices manufactured with these polymers.

The information provided in this manuscript may be of interest to researchers in biomedical and materials engineering, as well as to professionals linked to 3D printing applied to health. It is also of interest to the medical device manufacturing industry, sterilization companies and hospital biosafety managers, as well as regulatory bodies and health control agencies, which require clear evidence for the evaluation and approval of new technologies based on 3D-printed polymers.

Unlike previous studies that focused on single polymers or evaluated only mechanical degradation after sterilization, this work provides a comprehensive, cross-material comparison integrating mechanical, thermal, and chemical analyses for three widely used 3D-printed polymers (PLA, PLA + CF, and PETG). The use of standardized sterilization cycles, coupled with DSC–FTIR correlation and mechanical performance evaluation, enables a systematic interpretation of the relationship between molecular structure, crystallinity, and macroscopic behavior. This integrated approach contributes to a broader understanding of sterilization-induced transformations in FDM-printed polymers, establishing a framework that can guide material selection and qualification for reusable medical devices.

Section 2 of this document describes the materials addressed, sterilization methods, and analysis procedures used; Section 3 presents the results of mechanical and thermal tests; and Section 4 discusses the most relevant findings and proposes possible lines of future research.

## 2. Materials and Methods

### 2.1. Materials

**Figure 1:**
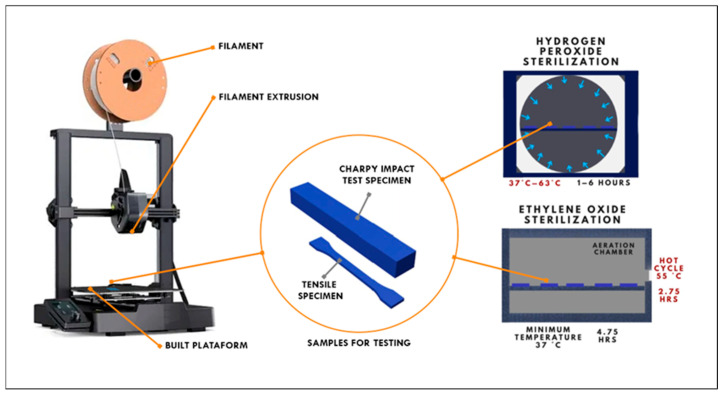
Schematic of the printing process and sterilization methods used.

The tests were conducted on three commercially available 3D-printed filaments: PETG (black, 1.75 mm diameter), PLA (red, 1.75 mm diameter), and PLA + FC (black, 1.75 mm diameter, 85% PLA) supplied by SUNLU [15], with a uniform diameter tolerance of ±0.02 mm. The general methodological process can be observed in Figure 1.

### 2.2. Preparation Processes

**Figure 2:**
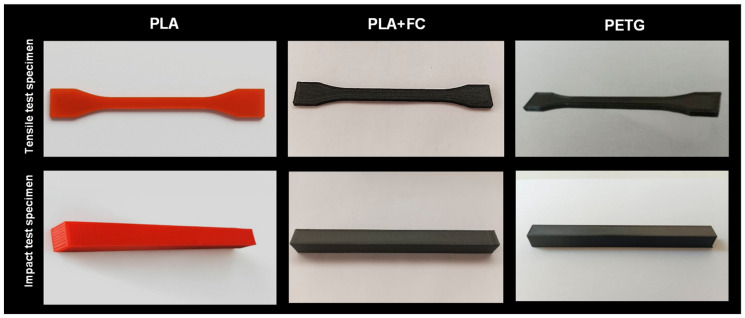
Tensile, impact, and hardness test specimens of PLA, PLA + FC, and PETG.

CAD software - SOLIDWORKS 2023 [16] was used to model the 3D-printed specimens for various tests, including tensile testing (dog-bone shape of ISO 527 standard [17]) and Charpy impact testing (rectangular prism). After modeling, the file was saved in STL format, and the G-code was generated using the free 3D-printing slicer software UltiMaker Cura 4.9. Finally, all samples were printed using the 3D printer Xinkebot Orca 2 Cygnus (Xinkebot, Shenzhen, China). The printing parameters were preconfigured in the mentioned slicer, with each material having its own specific printing characteristics, as detailed in Table 2. The three types of filaments tested (PETG, PLA, and PLA + FC) were printed with an 80% infill using the linear infill pattern from the UltiMaker Cura 4.9 software, as shown in Figure 2.

#### 2.2.1. Sterilization Processes

The sterilization conditions applied in this study were selected based on their clinical relevance and alignment with standard practices for medical device sterilization. Hydrogen peroxide vapor sterilization was performed at 30 °C for 60 min using a 50% H_2_O_2_ concentration, which corresponds to low-temperature plasma-based sterilization protocols widely used for polymeric medical devices. Ethylene oxide sterilization was conducted at 55 °C for 60 min followed by a 5 h aeration phase, consistent with ISO 11135 [18] recommendations and commonly applied in hospital and industrial sterilization processes for heat-sensitive materials. These parameters reflect real-world conditions intended to ensure sterility while minimizing thermal degradation of additive-manufactured components.

The sterilization conditions applied in this study were selected based on clinical relevance and international standards for medical device sterilization.

##### Hydrogen Peroxide (HP)

Vapor/plasma sterilization was carried out in a low-pressure chamber (Sterrad^®^ NX, Advanced Sterilization Products, Irvine, CA, USA) using a 50% H_2_O_2_ solution. The process took place at approximately 30 ± 2 °C under reduced pressure for 60 min, followed by a drying phase to remove residual peroxide. These parameters are consistent with ISO 22441:2022 [19], which specifies the requirements for vaporized hydrogen peroxide sterilization of medical devices. Each specimen was placed in a Tyvek^®^ sterilization pouch (DuPont, Wilmington, DE, USA) to ensure uniform gas exposure and prevent post-sterilization contamination.

##### Ethylene Oxide (EO)

Sterilization was conducted in a Steri-Vac™ 3M Gas Sterilizer/Aerator (3M, St. Paul, MN, USA) under the following conditions: EO gas concentration of 600 mg/L, temperature 55 ± 2 °C, relative humidity 60 ± 5%, and exposure time of 60 min. After exposure, an aeration phase of 5 h was applied at ambient pressure and temperature to ensure safe desorption of residual EO. The protocol followed the recommendations of ISO 11135:2014 [18] for EO sterilization of medical devices.

These specifications ensure reproducibility of the sterilization processes and allow assessment of their practical implications for the use of 3D-printed polymeric components in medical applications. The specimens were completely sealed to safeguard their properties.

In Table 2, the labels used for each material and its sterilization process are identified with the following abbreviations: SE: Non-Sterilized, EO: Ethylene Oxide Sterilization, HP: Hydrogen Peroxide Sterilization. Henceforth, these abbreviations will be used to identify the samples and their sterilization processes.

### 2.3. Tests

For each test, 21 specimens were printed: 7 SE, 7 HP, and 7 EO. All mechanical, thermal, and chemical characterizations were performed within 24 h after completing the sterilization processes. This approach aimed to minimize uncontrolled environmental influences, such as moisture uptake or thermal relaxation, ensuring that the measured properties reflected the immediate effects of sterilization

#### 2.3.1. Mechanical Characterization

##### Tensile Test

Tensile tests were performed after 3D printing and subsequent sterilization to evaluate the mechanical response of the materials. The experiments were conducted at room conditions (23 ± 2 °C, 50 ± 5% RH) using a SHIMADZU AGS-X [20]. universal testing machine (Shimadzu Corp., Kyoto, Japan) equipped with a 10 kN load cell, following the ISO 527-2:2012 [17] standard for plastics (type 1BA specimens). Each condition (Control, HP, and EO) was tested with five replicates (*n* = 5).

The test speed was 1 mm·min^−1^ for modulus determination, followed by 5 mm·min^−1^ for tensile strength and elongation at break. Recorded parameters included the elastic modulus (E), ultimate tensile strength (σₘ), and elongation at break (εᵦ). After testing, the fractured samples were preserved in sealed polyethylene bags to prevent surface contamination before subsequent fractographic analysis. 

##### Charpy Impact Test

The impact test was conducted after the samples were correctly printed and sterilized. The samples did not require notching. The tests were carried out according to the ASTM D-6110 [21] standard using an IBERTEST Charpy impact pendulum, impactest-50 (IBERTEST, Madrid, Spain) [22], at room temperature. For this test, the specimens were printed horizontally, and before entering the test area (15 °C), the dimensions of each sample were measured, and the test was conducted with a 50.03 J hammer, charpy impact pendulum IBERTEST Impactest-50 (IBERTEST, Madrid, Spain). Desired values such as impact resistance and absorbed energy were recorded, and the standard deviation was later calculated. The specimens were fully identified and sealed.

##### Fracture Surface Analysis

A fracture surface analysis was carried out based on the Charpy impact test using an stereomicroscope. The type of fracture (ductile or brittle), layer or perimeter separation, filament fusion, and changes between samples were reviewed. A magnification of 100× and white light were used.

##### Thermomechanical Analysis

In this study, a Q400 TMA thermomechanical analyzer (TA Instruments, Newcastle, DE, USA) was used to evaluate the thermal behavior of square specimens measuring 3 mm × 3 mm × 2 mm, with the aim of measuring changes in the glass transition temperature (Tg). The temperature range varied from room temperature (25 °C) to 130 °C for all samples, which were heated up to 200 °C. The heating rate was 5 °C/minute, in a nitrogen atmosphere at 50 mL/min. A preload of 0.02 N was applied, and a load of 0.005 N was maintained during the test.

##### Fourier Transform Infrared Spectroscopy

Fourier Transform Infrared Spectroscopy (FTIR) was performed to detect possible chemical changes in PLA, PLA + FC, and PETG after sterilization treatments with ethylene oxide (EO) and hydrogen peroxide (HP), compared to non-sterilized samples (SE). A Nicolet iS10 spectrometer (Thermo Scientific, Waltham, MA, USA) with a diamond ATR accessory was used, allowing direct analysis without prior sample preparation. Measurements were conducted in the 4000–400 cm^−1^ range, with a resolution of 4 cm^−1^ and 32 scans per spectrum. Environmental conditions were controlled, and a background scan was taken before each measurement. Changes in characteristic bands of functional groups such as carbonyls, esters, and C–O bonds were evaluated to identify potential degradation, oxidation, or crosslinking processes induced by sterilization.

##### Dynamic Mechanical Analysis

Dynamic mechanical analysis (DMA) experiments were carried out using an AR-G2 rheometer (TA Instruments, Newcastle, DE, USA) equipped with a single-cantilever solid-sample geometry. Rectangular specimens (40 × 10 × 3.5 mm) were tested under nitrogen flow (40 mL·min^−1^) in an Environmental Test Chamber (ETC) for precise temperature control. The measurements were performed in a temperature ramp mode from 25 to 150 °C at a heating rate of 3 °C·min^−1^, with a frequency of 1 Hz and strain amplitude of 0.05%. The applied axial force was automatically adjusted between 0.1 and 50 N to maintain contact throughout the test. Instrument calibration was verified prior to the experiments. The glass transition temperature (*Tg*) was determined from the peak of the loss tangent (tan δ) curve.

##### Differential Scanning Calorimetry

Differential scanning calorimetry (DSC) measurements were carried out using a DSC Q2000 (TA Instruments, New Castle, DE, USA) [23] equipped with a Standard Cell RC module. Samples of 0.7–1.8 mg were sealed in standard aluminum pans and analyzed under nitrogen flow (40 mL·min^−1^). The thermal program consisted of an equilibration at 40 °C followed by a heating ramp from 40 °C to 200 °C at a rate of 10 °C·min^−1^. Temperature calibration was verified using indium (156.6 °C), tin (231.9 °C), and zinc (419.5 °C) standards.

The glass transition temperature (*Tg*) was determined as the midpoint of the step change in the heat flow curve, while the cold crystallization (*Tcc*) and melting (*Tm*) temperatures were identified from the peak maxima of their respective transitions.

The degree of crystallinity (Xc) was calculated using the following Equation (1):

(1) $$X_{c}\left(\%\right)=\frac{\Delta H_{m}-\Delta H_{cc}}{\Delta H_{m}^{0}}\times 100$$

where $\Delta H_{m}$ is the melting enthalpy (J·g^−1^), $\Delta H_{cc}$ is the cold crystallization enthalpy (J·g^−1^), and $\Delta H_{m}^{0}$ is the enthalpy of 100% crystalline material, taken as 93 J·g^−1^ [24] for PLA and 40 J·g^−1^ for PETG [25], according to reported literature values. Although the DSC scans extended up to 500 °C, no evidence of decomposition was observed in the thermograms, as baseline stability and endothermic transitions remained consistent throughout the heating cycle. Therefore, the chosen temperature range did not compromise the accuracy of the thermal transition data.

### 2.4. Statistical Analysis

Statistical analyses were performed using OriginPro 2024 (OriginLab Corp., USA) [26]. Normality (Shapiro–Wilk) and homogeneity of variance (Levene) were first assessed. When both assumptions were met, a one-way ANOVA followed by Tukey’s post hoc test was applied. When variances were not homogeneous, Welch’s ANOVA with Games–Howell post hoc was used. If normality or homogeneity assumptions were not satisfied, Kruskal–Wallis tests with Dunn–Bonferroni post hoc were applied. Results are reported as mean ± standard deviation (*n* = 5), with effect sizes (η^2^) included when applicable. The raw data supporting this study are available in the supplementary material of Ref. [27].

## 3. Results

### 3.1. Mechanical Characterization

The following section emphasizes the critical role of 3D printing in medical applications, particularly how sterilization processes can impact the mechanical properties of 3D-printed materials. These impacts may include alterations in strength, stiffness, and other characteristics relevant to tensile testing.

**Figure 3:**
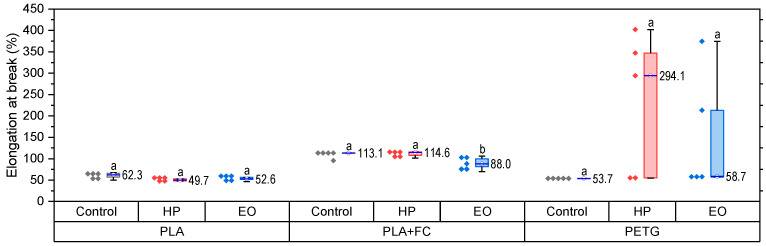
Elongation at break (%) of 3D-printed PLA, PLA + FC, and PETG samples after sterilization with hydrogen peroxide (HP) and ethylene oxide (EO), compared to control specimens. Different letters show statistically significant differences between formulations (*p* < 0.05).

**Figure 4:**
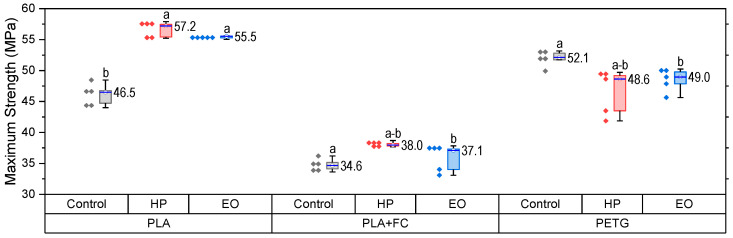
Maximum Strength (MPa) of 3D-printed PLA, PLA + FC, and PETG samples after sterilization with hydrogen peroxide (HP) and ethylene oxide (EO), compared to control specimens. Different letters show statistically significant differences between formulations (*p* < 0.05).

**Figure 5:**
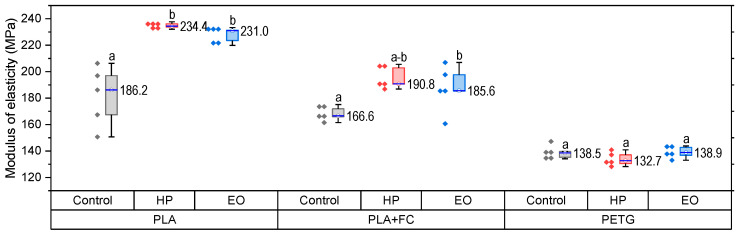
Modulus of elasticity (MPa) of 3D-printed PLA, PLA + FC, and PETG samples after sterilization with hydrogen peroxide (HP) and ethylene oxide (EO), compared to control specimens. Different letters show statistically significant differences between formulations (*p* < 0.05).

The tensile properties of PLA, PLA + FC, and PETG before and after sterilization are presented in Figure 3 (PLA), Figure 4 (PLA + FC), and Figure 5 (PETG).

The elongation at break results (Figure 3) demonstrate distinct responses of PLA, PLA + FC, and PETG to sterilization. For PLA, both HP and EO treatments caused the production of a slight decrease in ductility. PLA + FC maintained elongation levels after HP sterilization, while EO significantly decreased it to ~88%, indicating that carbon fiber reinforcement reduces but does not fully prevent the adverse effects of sterilization. For PETG, HP treatment produced a substantial increase in elongation (~294%), though with large variability (*p* > 0.05), whereas EO led to a pronounced decrease (~59%) compared to the control (~54%). These findings highlight that sterilization effects strongly depend on the polymer matrix, with PLA becoming less ductile, PLA + FC partially mitigating losses, and PETG exhibiting opposite responses under HP and EO treatments.

Statistical analysis confirmed that the PLA-FC Control and PETG-EO groups did not meet normality (Shapiro–Wilk, *p* < 0.05), and variances were unequal (Levene, *p* = 0.007); therefore, non-parametric Kruskal–Wallis tests were used. No statistically significant differences were found among treatments for PLA and PLA-FC (*p* > 0.05). For PETG, despite the apparent trends—an increase after HP and a decrease after EO—the high variability in the HP group prevented statistical significance (*p* > 0.05).

The maximum tensile strength results (Figure 4) revealed clear differences among the three materials after sterilization. For PLA, both HP and EO treatments increased UTS significantly (*p* < 0.05), reaching ~57 MPa and ~55 MPa, respectively, evidencing a notable strengthening effect. In PLA + FC, HP sterilization slightly enhanced UTS to ~38 MPa, while EO maintained values comparable to the control (~34 MPa), indicating that fiber reinforcement buffers the impact of sterilization. For PETG, both sterilization methods resulted in values close to the control (~52 MPa), suggesting minimal sensitivity of UTS to sterilization. These outcomes confirm that PLA is more prone to strength enhancement after sterilization, PLA + FC exhibits only minor changes, and PETG remains largely unaffected.

Data satisfied normality and homogeneity assumptions (Shapiro–Wilk and Levene, *p* > 0.05). One-way ANOVA revealed significant differences among groups (F(8,36) = 105.5, *p* < 0.0001). Tukey’s test confirmed that in PLA, both HP and EO increased UTS significantly compared with the control (*p* < 0.001). In PLA-FC, no differences among treatments were detected. In PETG, HP significantly reduced UTS compared with control and EO (*p* < 0.05).

The elastic modulus results (Figure 5) reveal a consistent tendency toward increased stiffness in PLA after sterilization. PLA-HP reached ~244 MPa and PLA-EO ~231 MPa, both considerably higher (*p* < 0.05) than the control (~186 MPa), confirming that sterilization promotes a stiffer but less ductile behavior. For PLA + FC, the control value of ~166 MPa increased slightly to ~190 MPa with HP and to ~185 MPa with EO, indicating only moderate changes due to the reinforcing effect of carbon fibers. In PETG, values remained close to the control (~135 MPa), evidencing negligible effects of sterilization on stiffness. These observations suggest that sterilization significantly modifies the stiffness of PLA, moderately affects PLA + FC, and has almost no impact on PETG. Statistical assumptions of normality were satisfied, whereas Levene’s test indicated unequal variances (*p* = 0.020). Accordingly, a Welch ANOVA was performed, revealing significant differences among groups (F(8,14.6) = 386.7, *p* < 0.0001). Games–Howell post hoc comparisons confirmed that PLA (HP and EO) exhibited significantly higher modulus values compared with PLA-FC and PETG (*p* < 0.001). No significant differences were found among the PETG groups.

**Figure 6:**
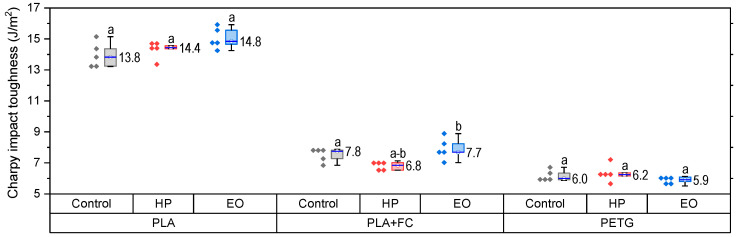
Change in the Charpy impact strength properties in PLA, PLA + FC and PETG 3D-printed samples. Different letters show statistically significant differences between formulations (*p* < 0.05).

The results of the Charpy impact test for PLA, PLA + FC, and PETG (Figure 6) show that there is no significant variation in toughness after applying the sterilization processes. This suggests that while tensile properties are sensitive to sterilization, the capacity to absorb energy during impact remains largely unchanged, which is relevant for medical applications requiring dimensional stability and toughness.

Normality and homogeneity assumptions were satisfied. ANOVA showed no significant differences for PLA (F(2,12) = 3.20, *p* = 0.077) or PETG (F(2,12) = 1.46, *p* = 0.271). In PLA-FC, however, significant differences were found (F(2,12) = 6.07, *p* = 0.015, η^2^ = 0.67), with Tukey’s test indicating that HP-treated samples had significantly lower impact resistance compared with the control (*p* < 0.05).

### 3.2. Microstructural Analysis

**Figure 7:**
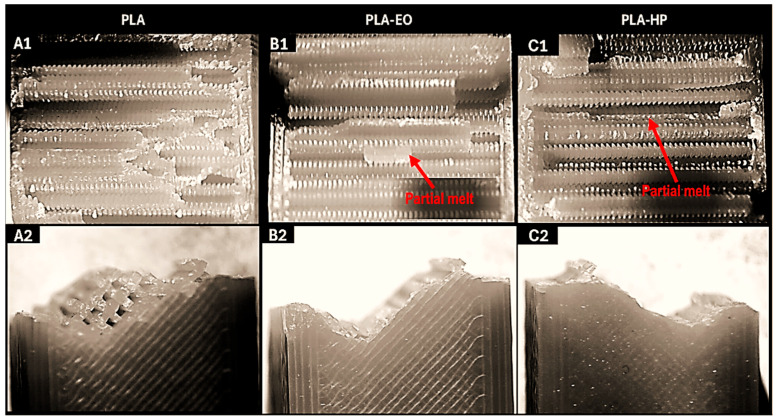
Cross-sectional (**A1**–**C1**) and lateral fracture images (**A2**–**C2**) from the Charpy test of PLA (Control), PLA-EO, PLA-HP.

**Figure 8:**
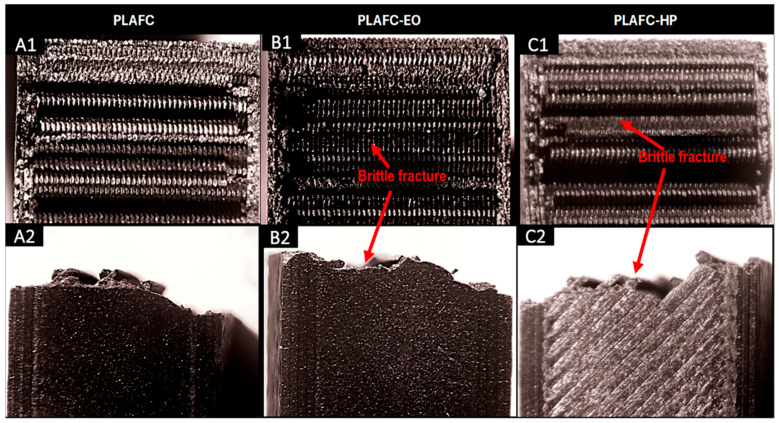
Cross-sectional (**A1**–**C1**) and lateral fracture images (**A2**–**C2**) from the Charpy test of PLA + FC (Control), PLA + FC-EO, and PLA + FC-HP.

**Figure 9:**
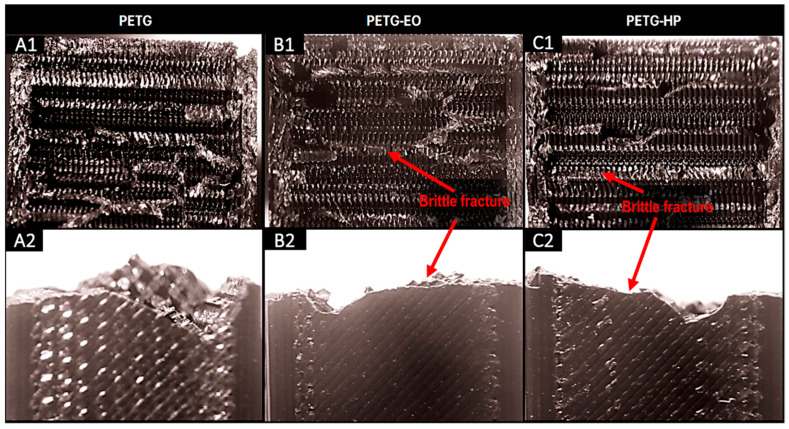
Cross-sectional (**A1**–**C1**) and lateral fracture images (**A2**–**C2**) from the Charpy test of PETG (Control), PETG-EO, PETG-HP.

The fracture surfaces of the control samples, along with those of the sterilized samples subjected to the Charpy impact test, were examined using a stereomicroscope. The results are shown in the corresponding Figures for PLA-based samples, PLA + FC, and PETG-based samples. For all samples (Figure 7, Figure 8 and Figure 9), a brittle fracture was observed, with no signs of delamination between the printed layers. Furthermore, detailed inspection revealed that crack initiation occurred at sharp geometrical discontinuities, especially at the junctions between infill pattern layers (anisotropic behavior), which acted as stress concentrators. These zones are identified in the stereomicrographs as the points of origin of fracture propagation, which extended rapidly across the cross-section following relatively straight paths. However, partial filament melt was noted in the PLA samples sterilized with HP and EO (Figure 7).

### 3.3. Thermomechanical Analysis

The following section presents the behavior of materials during the thermal test ‘TMA Thermomechanical Analyzer’ to determine variations in the glass transition temperature and thermal expansion coefficients of the three FDM-printed materials.

**Figure 10:**
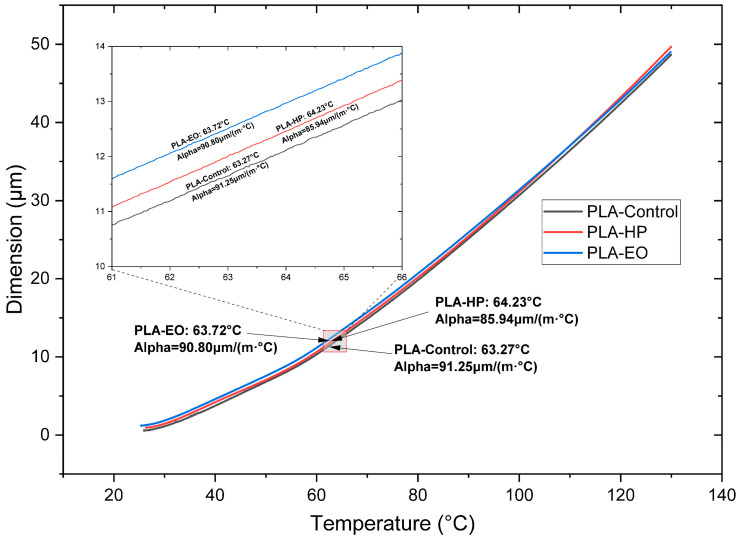
Thermomechanical Analysis Curves of 3D-Printed PLA (FDM) Sterilized with Hydrogen Peroxide (HP) and Ethylene Oxide (EO). The dimensional change (µm) indicates the difference in size of the sample at temperature T compared to room temperature.

**Figure 11:**
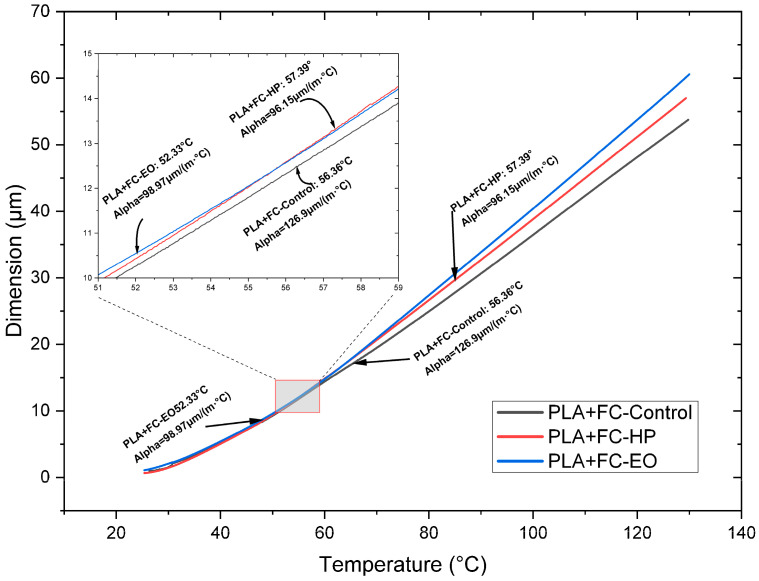
Thermomechanical Analysis Curves of 3D-printed (FDM) PLA + FC sterilized with hydrogen peroxide (HP) and ethylene oxide (EO). The dimensional change (µm) indicates the size difference in the sample at temperature compared to room temperature.

**Figure 12:**
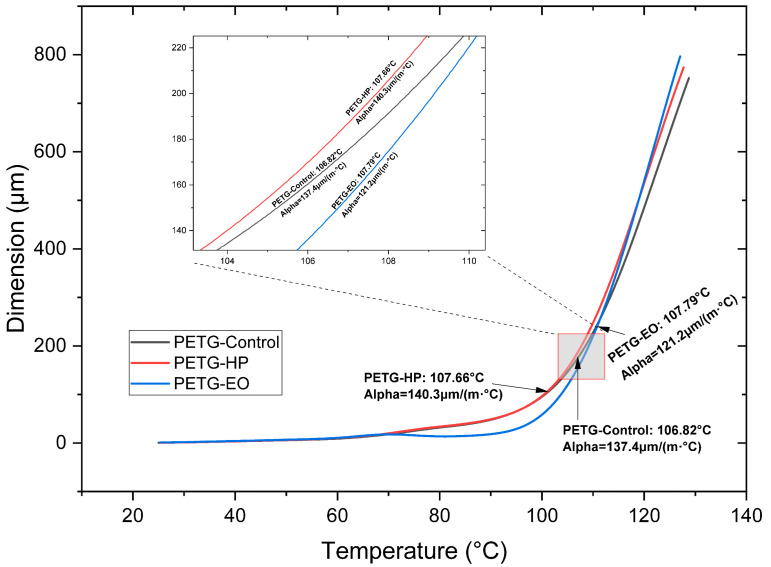
Thermomechanical Analysis Curves of 3D-printed (FDM) PETG sterilized with hydrogen peroxide (HP) and ethylene oxide (EO). The dimension change (µm) indicates the size difference in the sample at temperature T compared to room temperature.

The thermal response of the samples is shown in Figure 10, Figure 11 and Figure 12. HP sterilization slightly increased Tg for PLA and PETG, while EO reduced Tg for PLA + FC by approximately 4 °C (Table 3). These trends suggest that HP promotes localized structural stabilization, whereas EO may induce hydrolytic interactions at the polymer–fiber interface.

In the TMA test with PLA, no significant variation in the CTE at low and high temperatures is identified (Figure 10 and Table 3) when sterilized with HP or EO, compared to the SE sample. On the other hand, in PLA + FC (Figure 11 and Table 3), after HP and EO sterilization, a decrease of approximately 30.0 µm is observed at low temperatures. At high temperatures, the CTE of PLA + FC shows an increase of 26.6 µm after the HP sterilization process.

When analyzing the CTE at low temperatures in PETG (Figure 12 and Table 3) and comparing it with the SE sample, the value slightly increases with the HP sterilization process and decreases by 16.2 µm with the EO process. Regarding the CTE at high temperatures, a value of 10,324 µm is identified in the SE sample. However, after the EO sterilization, the CTE reaches 11.8 µm, which is the highest value observed.

### 3.4. Dynamic Mechanical Analysis

The present analysis examines the viscoelastic behavior of the materials using Dynamic Mechanical Analysis (DMA), aiming to determine the storage modulus and damping factor (tan δ), as well as to identify the glass transition temperature (Tg) of the three FDM-printed polymers. The DMA curves and corresponding data for PLA, PLA + FC, and PETG are presented in the following tables and figures, allowing the evaluation of possible modifications induced by the sterilization processes.

Sterilization slightly affected the glass transition temperatures of the polymers. PLA showed a minor decrease with HP and an increase with EO, while PLA + FC exhibited minimal changes, indicating higher stability (Table 4). PETG was the most affected, with notable Tg increases after both treatments. Overall, the results suggest that PETG is more sensitive to sterilization, whereas PLA + FC maintains stable thermal behavior.

**Figure 13:**
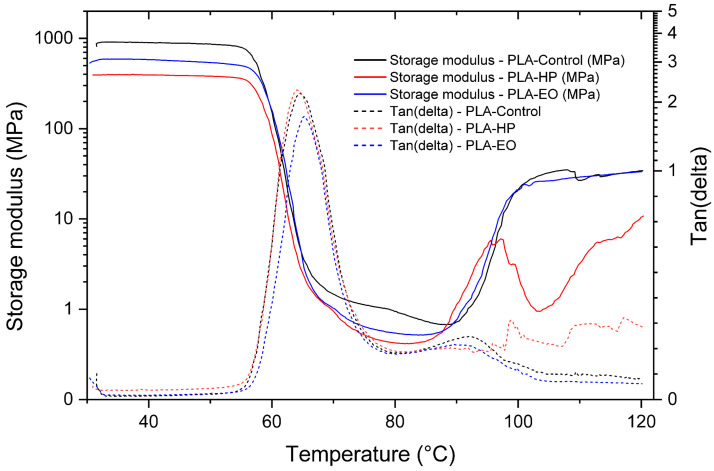
Dynamic Mechanical Analysis (DMA) results of 3D-printed (FDM) PLA, showing storage modulus and tan δ for samples sterilized with hydrogen peroxide (HP) and ethylene oxide (EO), compared to the control.

The control sample exhibited the typical profile of PLA (Figure 13), with a high storage modulus in the glassy region, followed by a sharp decrease around the glass transition (≈60–70 °C) and a well-defined tan δ peak (Tg = 64.58 °C). In contrast, PLA sterilized with hydrogen peroxide showed the most pronounced changes, including a reduced modulus, a broader tan δ peak, and an anomalous recovery of stiffness above 90 °C, with a slightly lower Tg (64.05 °C), suggesting structural reorganization or recrystallization. PLA exposed to ethylene oxide presented a more moderate effect, maintaining a general curve shape similar to the control but with a consistently lower modulus and a slightly higher Tg (65.25 °C). These results indicate that hydrogen peroxide induces stronger alterations in the molecular mobility and thermal–mechanical stability of PLA, while ethylene oxide preserves its dynamic mechanical response to a greater extent.

**Figure 14:**
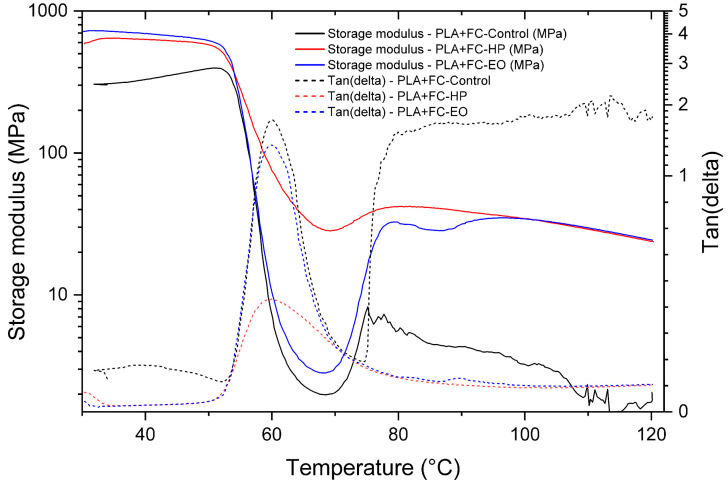
Dynamic Mechanical Analysis (DMA) results of 3D-printed (FDM) PLA + FC, showing storage modulus and tan δ for samples sterilized with hydrogen peroxide (HP) and ethylene oxide (EO), compared to the control.

The PLA + FC control sample (Figure 14) exhibited a pronounced decrease in E′ around 60 °C, corresponding to the glass transition region (Tg = 60.16 °C), together with a broader tan δ peak, indicative of higher chain mobility. In contrast, samples sterilized with hydrogen peroxide (HP, Tg = 59.97 °C) and ethylene oxide (EO, Tg = 59.7 °C) showed narrower and less intense tan δ peaks, suggesting restricted molecular motion. Both sterilization treatments maintained higher storage modulus values above Tg compared to the control, evidencing improved mechanical stability in the rubbery state. EO treatment induced a slightly lower damping capacity. These results demonstrate that sterilization processes can alter the viscoelastic performance of PLA + FC composites. Overall, the treatments appear to enhance stiffness at elevated temperatures while reducing energy dissipation capacity.

**Figure 15:**
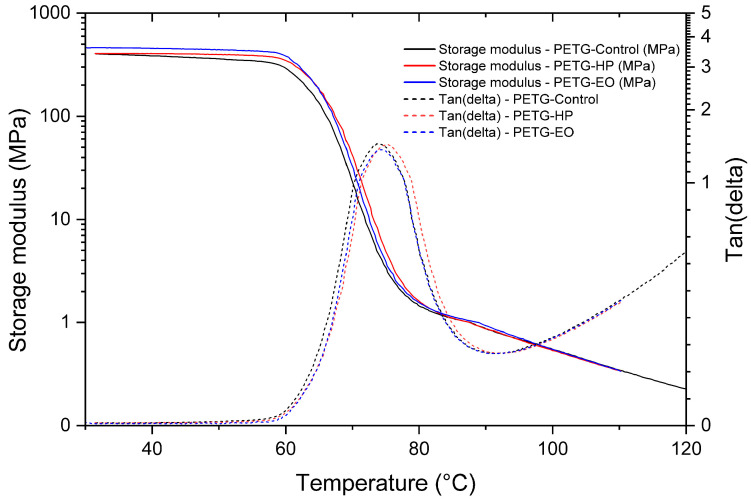
Dynamic Mechanical Analysis (DMA) results of 3D-printed (FDM) PETG, showing storage modulus and tan δ for samples sterilized with hydrogen peroxide (HP) and ethylene oxide (EO), compared to the control.

The DMA results of PETG samples (Figure 15) revealed that the storage modulus remained stable up to approximately 65 °C, after which a sharp decrease indicated the glass transition. The control, HP-sterilized, and EO-sterilized specimens showed nearly overlapping curves, demonstrating that sterilization did not significantly alter the initial stiffness of the polymer. The tan(δ) profiles exhibited clear peaks corresponding to the Tg, located at 73.64 °C for the control, 75.35 °C for HP, and 74.35 °C for EO samples. These subtle differences suggest minor changes in molecular mobility but no substantial modification of the viscoelastic behavior. In summary, PETG exhibited high thermal and mechanical stability after sterilization treatments.

### 3.5. Differential Scanning Calorimetry

#### Differential Scanning Calorimetry

**Figure 16:**
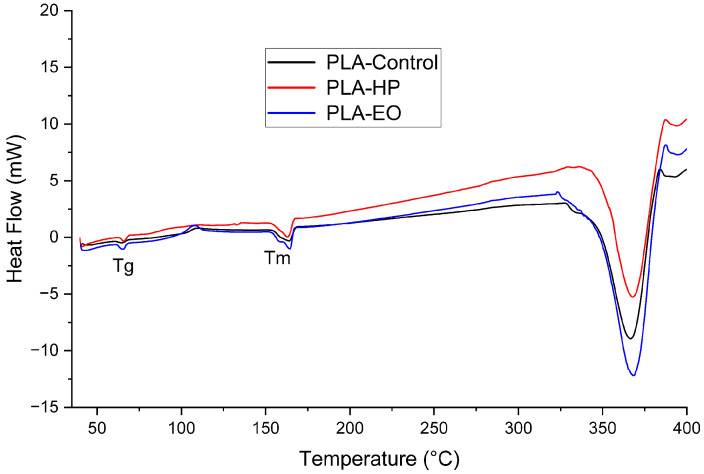
Differential Scanning Calorimetry (DSC) curves of 3D-printed (FDM) PLA sterilized with hydrogen peroxide (HP) and ethylene oxide (EO), compared to the control sample.

**Figure 17:**
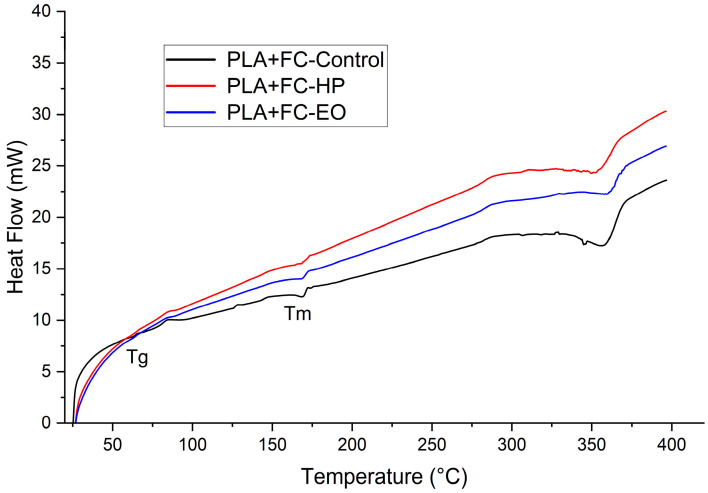
Differential Scanning Calorimetry (DSC) curves of 3D-printed (FDM) PLA + FC sterilized with hydrogen peroxide (HP) and ethylene oxide (EO), compared to the control sample.

**Figure 18:**
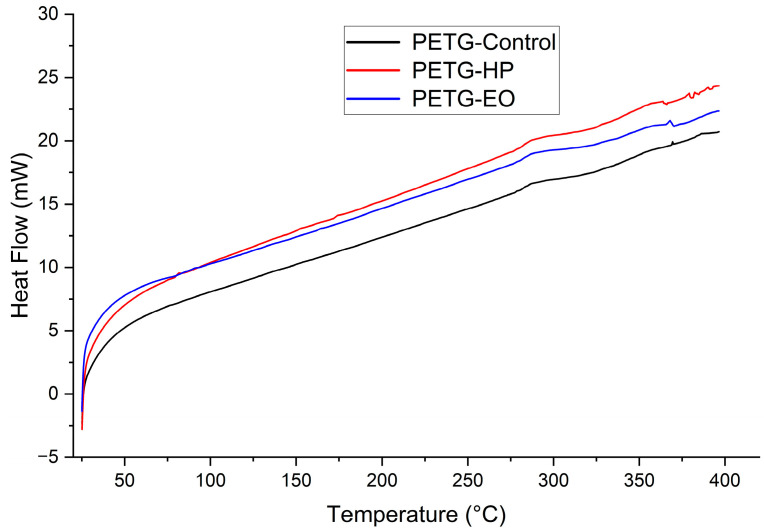
Differential Scanning Calorimetry (DSC) curves of 3D-printed (FDM) PETG sterilized with hydrogen peroxide (HP) and ethylene oxide (EO), compared to the control sample.

The DSC results (Table 5) and corresponding thermograms (Figure 16, Figure 17 and Figure 18) show that PLA and PLA + CF exhibited well-defined glass transition (Tg) and melting (Tm) temperatures, which remained stable after both sterilization treatments (ΔT < 2 °C). However, variations in crystallinity were observed. For PLA, the degree of crystallinity (Xc) increased markedly after HP sterilization (from 16.6% to 36.1%), suggesting recrystallization of amorphous domains induced by the oxidative environment of the process. EO sterilization caused a moderate increase in Xc (22.3%). These differences are consistent with the higher heat flow observed in the PLA-HP thermogram.

In the PLA + CF composite, Xc variations were smaller (9.5–16.7%), indicating that the carbon fiber reinforcement restricted molecular mobility and reduced recrystallization, thereby enhancing dimensional and thermal stability. The thermograms confirm this behavior, as the HP-treated composite shows a slightly higher heat flow compared with the control, while EO produces a less pronounced change. This stability aligns with the mechanical data, where the composite exhibited less variation in stiffness and strength after sterilization.

PETG, being an amorphous copolymer, presented a broad and weak glass transition near 80 °C without a detectable melting peak, consistent with its low crystallinity and absence of long-range order. The PETG-HP and PETG-EO curves show a gradual increase in heat flow with temperature, suggesting improved energy absorption capacity but no significant structural rearrangement.

Overall, these results indicate that sterilization primarily affects the crystalline morphology of PLA-based materials, particularly under HP treatment, while PETG remains largely unaffected. Both HP and EO processes preserved the main thermal transitions and did not compromise the thermal stability of the polymers.

### 3.6. Fourier Transform Infrared Spectroscopy

This section analyzes the chemical behavior of the materials using Fourier Transform Infrared Spectroscopy (FTIR), aiming to identify characteristic absorption bands and detect any chemical modifications in the three FDM-printed polymers. To interpret the observed bands, typical wavenumber ranges corresponding to organic functional groups will be considered, based on reference tables and graphs from preliminary studies of organic compounds [25].

The FTIR spectra and corresponding data for PLA, PLA + FC, and PETG are presented in the following tables and figures.

**Figure 19:**
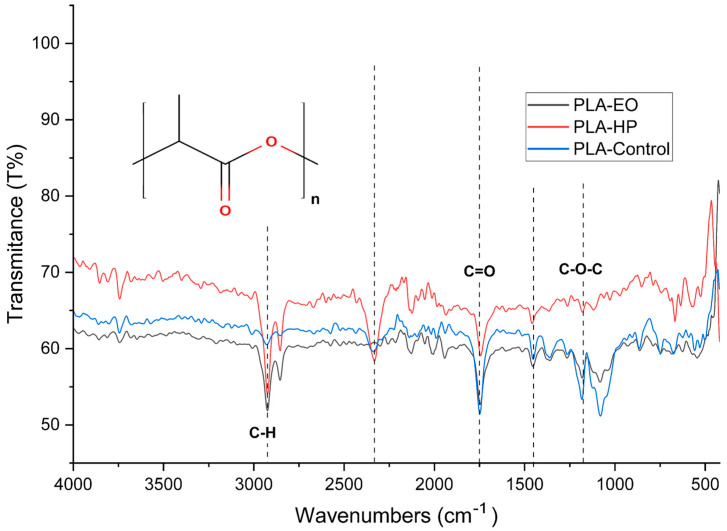
Fourier Transform Infrared Spectroscopy Curves of 3D-printed (FDM) PLA sterilized with hydrogen peroxide (HP) and ethylene oxide (EO).

The FTIR spectrum of untreated PLA (Figure 19) shows distinctive vibrational bands that are characteristic of its molecular structure. A prominent absorption around 1750 cm^−1^ corresponds to the stretching vibration of carbonyl groups (C=O) in ester linkages, which serves as a clear fingerprint of the polyester backbone [1]. In the region between 1180 and 1080 cm^−1^, the spectrum exhibits asymmetric and symmetric stretching bands of C–O–C, further confirming the presence of ester-type functionalities. Additionally, a band near 1450 cm^−1^ is attributed to the bending vibrations of methyl groups (CH_3_). The C–H stretching vibrations appear around 2995 and 2945 cm^−1^, associated with the methine and methyl groups in the polymer chain. Finally, the region between 875 and 750 cm^−1^ contains out-of-plane vibrations that often reflect the crystalline or amorphous morphology of the polymer [25]. Taken together, these spectral features confirm the presence of lactide repeating units and suggest a semi-crystalline nature in the PLA sample [25].

Following hydrogen peroxide (HP) sterilization, notable spectral changes are observed. The intensity of the carbonyl stretching band at 1750 cm^−1^ is reduced, which may indicate partial hydrolysis of the ester bonds or oxidative scission of the polymer chain [28]. This observation is consistent with known mechanisms of HP-induced degradation, in which hydroxyl radicals attack ester linkages, causing chain scission and the formation of terminal hydroxyl or carboxyl groups. A slight decrease in the intensity of the C–O–C stretching bands in the 1180–1080 cm^−1^ range supports the hypothesis of ester bond cleavage, implying a loss of structural integrity. Additionally, slight shifts and broadening of the CH_3_ bending and C–H stretching bands (~1450 and ~2945 cm^−1^) may suggest conformational disorder or changes in the crystalline-to-amorphous phase ratio due to surface oxidation [29]. Overall, HP treatment appears to significantly affect the ester structure in PLA, potentially reducing molecular weight and mechanical strength, although it may enhance surface hydrophilicity [28].

In contrast, the FTIR spectrum of PLA sterilized with ethylene oxide (EO) exhibits much subtler alterations. The carbonyl stretching band at 1750 cm^−1^ remains virtually unchanged, indicating that the ester bonds are preserved and that the polymer backbone undergoes little to no scission. The intensities of the CH and CH_3_ bands (~2945 and 1450 cm^−1^) remain stable, suggesting that no major conformational or structural changes occur. Furthermore, the absence of new absorption bands or significant broadening indicates that PLA is chemically inert to EO under standard sterilization conditions. This minimal reactivity makes EO a more conservative and suitable method for sterilizing PLA, especially in applications where preserving the polymer’s chemical and mechanical integrity is critical.

**Figure 20:**
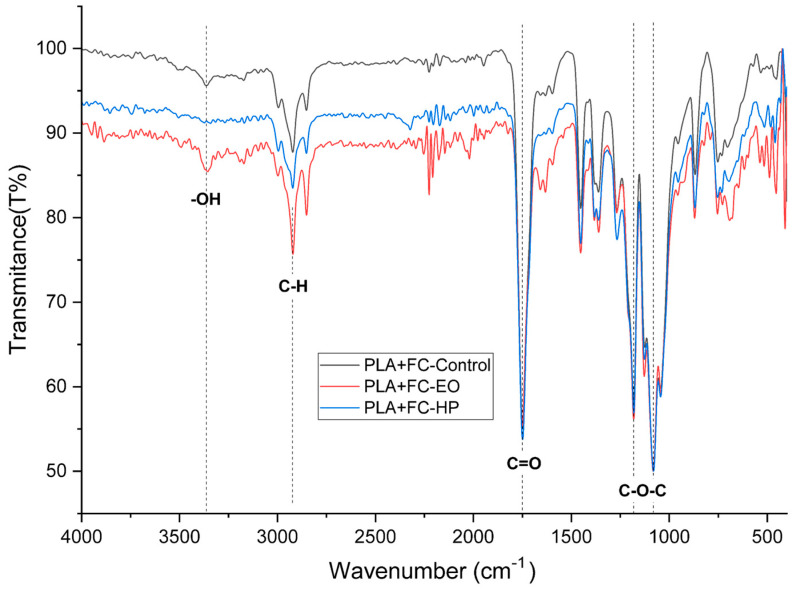
Fourier Transform Infrared Spectroscopy Curves of 3D-printed (FDM) PLA + FC sterilized with hydrogen peroxide (HP) and ethylene oxide (EO).

The FTIR spectrum of untreated PLA + CF (Figure 20) exhibited characteristic peaks corresponding to polylactic acid functional groups. A strong absorption band was observed at 1747 cm^−1^, attributed to the ester C=O stretching (C=O), a primary indicator of the PLA backbone. The asymmetric and symmetric CH_3_ and CH_2_ stretching vibrations appeared between 2995 and 2850 cm^−1^, while the C–O–C stretching modes were present in the 1180–1080 cm^−1^ region. Minor features were noted at 1454 and 1360 cm^−1^, related to bending vibrations of CH groups [30].

After HP sterilization, several changes were evident. The C=O stretching band at 1747 cm^−1^ showed a slight reduction in intensity, potentially indicating oxidative degradation or partial ester bond cleavage. The OH stretching region (broad band around 3500–3200 cm^−1^) became more prominent, suggesting the formation of hydroxyl end groups due to hydrolytic chain scission. Additionally, a notable decrease in the C–H stretching intensity (2850–2995 cm^−1^) was observed, indicating oxidation or chain scission of aliphatic segments. Minor changes were also seen in the C–O–C region, pointing to rearrangement or cleavage of ester linkages [31].

In the case of EO sterilization, spectral changes were less pronounced. The (C=O) band at 1747 cm^−1^ remained stable in intensity and position, suggesting minimal disruption to the ester groups. However, a slight increase in the OH region was observed, indicating some minor hydrolysis. The C–H stretching region remained mostly unchanged, while the C–O stretching bands displayed marginal shifts, possibly due to mild alkylation or surface-level interaction with EO [32].

**Figure 21:**
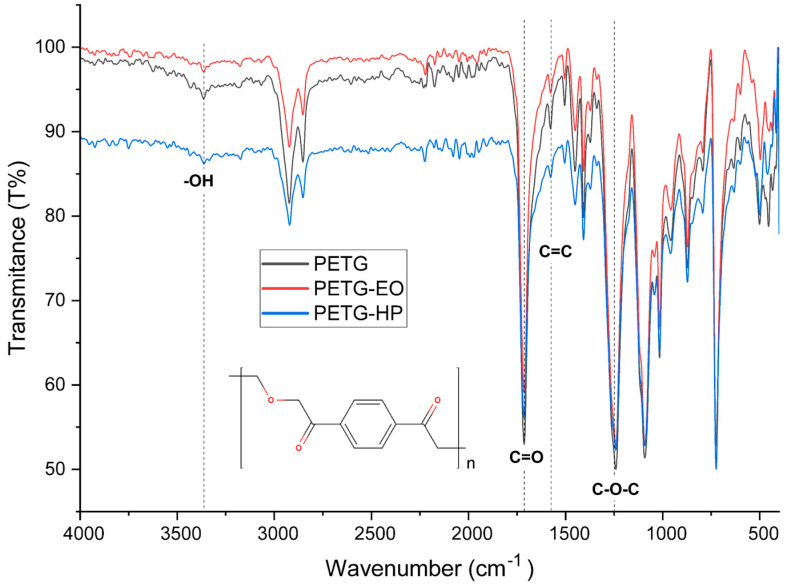
Fourier Transform Infrared Spectroscopy Curves of 3D-printed (FDM) PETG sterilized with hydrogen peroxide (HP) and ethylene oxide (EO).

The FTIR spectrum of untreated PETG (Figure 21) exhibits distinct absorption bands that are characteristic of its ester and glycol components. A prominent absorption band appears at 1712 cm^−1^, which is attributed to the stretching vibration of ester carbonyl groups (C=O). Additionally, aromatic skeletal vibrations, arising from the benzene rings in the terephthalate structure, are observed at 1578 cm^−1^ and 1505 cm^−1^ (C=C). The spectrum also displays C–O–C asymmetric and symmetric stretching vibrations at 1240 cm^−1^ and 1090 cm^−1^, respectively. Aliphatic chain mobility is evidenced by CH_2_ wagging and twisting vibrations at cm^−1^. A broad band in the region of 3300–3500 cm^−1^ is present, likely associated with terminal hydroxyl (–OH) groups or with small amounts of moisture absorbed on the polymer surface, which is expected given the notably hygroscopic nature of PETG [33].

Following sterilization with hydrogen peroxide (HP), several spectral changes are evident. The carbonyl band at 1712 cm^−1^ shows a marked increase in intensity, suggesting oxidative cleavage of ester bonds and the potential formation of carboxylic acid end groups. This treatment also results in a slight enhancement in the –OH stretching region (3300–3500 cm^−1^), which may be indicative of the formation of hydroxylated degradation products. Moreover, the C–O–C stretching bands at 1240 cm^−1^ and 1090 cm^−1^ exhibit a minor reduction in intensity, likely due to scission or rearrangement of the glycol components. Subtle shifts or changes in the intensity of the aliphatic deformation bands around 870 cm^−1^ imply molecular reorganization or alterations in the crystallinity of the polymer chains [34].

In contrast, the effects of ethylene oxide (EO) sterilization are more moderate. The spectrum shows slight increases in the intensity of both the carbonyl and C–O–C bands, though to a lesser extent than observed with HP treatment. The broad OH region (3300–3500 cm^−1^) remains largely unchanged, suggesting minimal hydrolytic or oxidative activity. Notably, the aromatic bands at 1578 cm^−1^ and 1505 cm^−1^ remain stable, indicating that the aromatic backbone of PETG is not compromised by EO exposure. Minor reductions in the intensity of the CH_2_ deformation bands near 870 cm^−1^ may point to superficial interactions or subtle modifications in chain mobility, but overall structural integrity appears to be preserved [35].

## 4. Discussion

The study aimed to evaluate the impact of sterilization processes, specifically hydrogen peroxide (HP) and ethylene oxide (EO), on the physical properties of three 3D-printed polymers: PLA, PLA + FC, and PETG. These materials were chosen due to their potential use in medical applications where sterilization is a crucial step. Given that interactions between polymer and sterilant are governed by molecular structure and morphology, we also outline how PLA (aliphatic semi-crystalline polyester), PLA + FC (fiber-restricted diffusion/compliance), and amorphous PETG (hygroscopic, aromatic-containing polyester) differentially interact with HP/EO and moisture, which explains why PLA tends to stiffen with reduced ductility, PLA + FC shows attenuated effects due to fiber-limited diffusion, and PETG exhibits only minor changes with near-control UTS and modulus.

This study focused on a fixed print orientation (linear infill pattern). However, due to the anisotropic nature of FDM, future research should consider how build orientation and raster angle may affect mechanical performance post-sterilization.

As shown in Section 3.1 the tensile test results reveal distinct behaviors across the three materials. PLA, known for its brittleness [36], showed a decrease in elongation at break after sterilization with both HP and EO. There was an increase in the modulus of elasticity and ultimate tensile strength, particularly with HP sterilization. This indicates that while PLA becomes less ductile, it gains stiffness and strength after sterilization. Such changes are supported by the FTIR results discussed in Section 3.4, which show that hydrogen peroxide (HP) treatment reduces the intensity of the carbonyl (C=O) and C–O–C ester bands, indicating chain scission and partial hydrolysis of ester bonds. This structural reorganization can promote crystallization, such behavior aligns with previous findings reported for this material [25,37]. This interpretation is further reinforced by DSC data, where PLA exhibited a marked increase in crystallinity after HP sterilization (from 16.6% to 36.1%)

In contrast, PLA + FC, which incorporates carbon fibers, exhibited a minimal change in elongation at break with HP sterilization but a notable reduction with EO, as shown in Figure 4. The modulus of elasticity and tensile strength saw slight increases, suggesting that carbon fibers provide some stabilization against the effects of sterilization. This stabilization may be attributed to the fact that carbon fibers serve as nucleating sites for PLA, accelerating crystallization, which enhances stiffness and heat resistance [38]. This interpretation is corroborated by the DSC analysis, where PLA + FC exhibited a slight increase in crystallinity (~5%). Nevertheless, EO still negatively impacts the ductility of the material.

PETG showed the highest elongation at break after sterilization, evidencing superior flexibility vs. PLA and PLA + FC, in line with [37]. Although tensile strength appears slightly lower after treatment, the decrease is not statistically significant (one-way ANOVA; values remain close to control). Minor surface oxidation/hydrolysis and moisture uptake in this hygroscopic polyester can slightly weaken intermolecular interactions and interlayer adhesion in FDM parts, signals that align with subtle FTIR changes (C=O/O–H after HP as shown in Section 3.6) and modest shifts in amorphous mobility (TMA/DMA). Consequently, both modulus and UTS remain effectively unchanged, confirming that PETG retains its mechanical integrity.

As shown in Section 3.1 and Figure 6, Charpy impact test results indicated that the sterilization processes did not significantly affect the toughness of any of the materials. This suggests that while tensile properties might be influenced by sterilization, the ability of these materials to absorb impact energy remains relatively stable. This is crucial for applications where impact resistance is necessary, such as in certain medical devices [38].

The microstructural analysis revealed brittle fractures in all materials, with some partial filament melting in PLA samples subjected to both HP and EO sterilization. This melting could be attributed to the sterilization processes leading to a loss of structural integrity at certain points. PLA + FC and PETG showed similar brittle fracture patterns, but the carbon fibers in PLA + FC may have mitigated some of the thermal effects, as there was less evidence of filament melting compared to PLA. With these results, we could use elements made from polymers like PLA sterilized with HP and EO in bone defect substitution applications [39], as the increase in mechanical properties such as Young’s modulus or UTS does not affect its function.

The thermomechanical analysis (Section 3.3) indicated slight increases in the glass transition temperature (Tg) for all materials after HP sterilization. This increase suggests that the sterilization process might enhance the thermal stability of these polymers to some extent. However, PLA + FC showed a decrease in Tg with EO sterilization, indicating a potential reduction in thermal stability [40,41]. These trends were further corroborated by DMA, which, being more precise for defining Tg, revealed consistent shifts with similar directionality, particularly confirming the reduction in PLA + FC with EO and the stabilization effect of HP in PLA and PETG. Likewise, DSC analysis supported these findings, showing comparable Tg values and, in addition, highlighting changes in the degree of crystallinity, most notably the increase observed in PLA after HP sterilization, which may explain the higher stiffness identified mechanically. FTIR spectrum, revealed a slight increase in the O–H stretching region (~3500–3200 cm^−1^) and marginal shifts in the C–O bands. These changes suggest mild hydrolysis or surface interactions that could have disrupted intermolecular forces resulting in a reduced glass transition temperature, which could be problematic for applications requiring high-temperature resistance.

The coefficient of thermal expansion (CTE) also varied among the materials. PLA + FC showed a significant decrease in CTE at low temperatures after both sterilization processes, indicating reduced dimensional stability. PETG, on the other hand, exhibited an increase in CTE at high temperatures with EO sterilization, this behavior can be explained by the chemical modifications observed in the FTIR spectrum, where slight increases in the intensity of the carbonyl (C=O) and C–O–C bands suggest mild oxidative or alkylation processes. Although the aromatic structure of PETG remains stable, these subtle chemical alterations may lead to increased molecular mobility or a reduction in intermolecular interactions at elevated temperatures. Consequently, the polymer chains could expand more freely under thermal stress, resulting in a higher coefficient of thermal expansion. A similar increase in thermal expansion has been reported in prior studies, where thermal sterilization-induced partial chemical degradation in PETG, enhancing molecular mobility and leading to greater dimensional instability [6]. These findings highlight the need for careful consideration of sterilization effects in applications where dimensional stability at elevated temperatures is critical. This behavior is consistent with previous studies, where thermal sterilization methods significantly increased the coefficient of thermal expansion in PETG due to partial chemical degradation and enhanced molecular mobility [38].

In PLA + FC, the carbon fibers likely limit chain mobility changes and act as barriers to oxidation, explaining the minor variations in mechanical properties. For PETG, the marked increase in elongation at break after HP treatment suggests reduced intermolecular interactions, possibly due to partial depolymerization and amorphous phase relaxation, while the chemical structure (with aromatic rings) remains stable as indicated by FTIR.

EO sterilization exhibited more conservative effects, consistent with its milder reactivity. Minimal FTIR changes and negligible variations in Tg and modulus support that EO does not significantly induce chain scission or crosslinking. However, slight decreases in Tg for PLA + FC could reflect subtle hydrolytic reactions at the polymer–fiber interface, reducing intermolecular cohesion. Overall, the combined influence of chain scission, potential crystallinity changes, and minor oxidative modifications explains the mechanical strengthening and reduced ductility in PLA, the stability in PLA + FC, and the flexibility increase in PETG after sterilization. 

These interpretations are supported by the FTIR findings, which showed a decrease in the C=O and C–O–C bands and the presence of hydroxyl-related signals after HP sterilization, confirming oxidative chain scission. The slight Tg increase observed in PLA and PETG after HP treatment indicates restricted segmental mobility, likely associated with secondary crystallization or localized densification, whereas the Tg decrease in PLA + FC after EO suggests minor hydrolytic reactions at the fiber–matrix interface. Similarly, the increase in CTE at high temperatures for PETG after EO points to enhanced molecular mobility, consistent with the subtle chemical modifications observed in its FTIR spectrum.

These mechanistic interpretations align with previous reports on oxidative degradation and structural rearrangements in polyesters subjected to sterilization [28,29,37,40].

Despite the consistency among the mechanical, thermal, and spectroscopic results, this study is limited by the absence of molecular-level analyses such as Gel Permeation Chromatography (GPC) or X-ray Diffraction (XRD). These techniques could quantitatively confirm the proposed mechanisms of chain scission, oxidative rearrangement, and recrystallization suggested by FTIR and DSC. Future work should therefore incorporate GPC to assess molecular weight distribution, XRD to characterize crystallite morphology, and surface analysis (AFM or SEM-EDX) to evaluate oxidation gradients. These additions would enhance the mechanistic depth and enable predictive modeling of polymer behavior under sterilization stress.

Finally, the development of new polymer blends or composites that combine the desirable properties of these materials while mitigating the adverse effects of sterilization could be a promising avenue for enhancing the mechanical and thermal performance of 3D-printed medical devices with PLA, PLA + FC and PETG.

## 5. Conclusions

In the mechanical characterization of the printed materials, it was identified that the increase in stiffness and strength (+22%) of PLA after sterilization could be advantageous in applications that require load-bearing capabilities, but the reduction in its ductility might limit its use in flexible devices. The behavior of PLA + FC suggests that, although carbon fibers provide some resistance to the effects of sterilization, ethylene oxide (EO) sterilization could still compromise its ductility, which necessitates careful consideration in applications where flexibility is critical. The robust performance of PETG throughout the tests suggests that it may be more suitable for a wider range of medical applications where both flexibility and sterilization are required. To minimize risk and ensure safety in a medical environment where 3D-printed parts with customized applications are used, the 3D-printed materials are sterilized with hydrogen peroxide (HP) and ethylene oxide (EO).

The thermal analysis revealed that sterilization slightly increased the glass transition temperature (Tg) of PLA and PETG, suggesting improved thermal stability. However, PLA + FC exhibited a decrease in Tg after EO sterilization, indicating potential thermal instability. The coefficient of thermal expansion (CTE) varied among the materials, with PLA + FC showing reduced dimensional stability at low temperatures, and PETG exhibiting increased CTE at high temperatures after EO sterilization.

In summary, hydrogen peroxide and ethylene oxide sterilization produced polymer-specific effects that can now be correlated with their intrinsic molecular morphology. HP treatment promoted chain scission and recrystallization in PLA, leading to stiffness enhancement; EO caused milder surface oxidation; and PETG remained largely stable due to its amorphous aromatic structure. These findings establish a structure–property–sterilization correlation useful for predicting polymer response in low-temperature sterilization environments. Practically, the results support the safe clinical reuse of 3D-printed PLA, PLA + CF, and PETG components under validated HP or EO cycles, provided that dimensional and mechanical constraints are respected. This framework can guide the qualification of additively manufactured materials for medical applications and future regulatory assessments.

## Figures and Tables

**Table 2:** Printing Parameters and Sterilization Process.

Item	Printing Temperature [°C]	Print Bed Temperature [°C]	Sterilization Process
PETG-Control	230	70	-
PETG-HP	230	70	HP
PETG-EO	230	70	EO
PLA + FC-Control	210	60	-
PLA + FC-HP	210	60	HP
PLA + FC-EO	210	60	EO
PLA-Control	200	60	-
PLA-HP	200	60	HP
PLA-EO	200	60	EO

Control refers to samples without sterilization, HP indicates sterilization with hydrogen peroxide, and EO indicates sterilization with ethylene oxide.

**Table 3:** Glass transition temperature (Tg) and coefficient of thermal expansion—CTE (CTE1 represents the low-temperature, and CTE2 represents the high-temperature) after TMA testing on 3D-printed samples (PLA, PLA + FC and PETG) sterilized by HP and EO.

	PLA			PLA + FC			PETG		
	SE	HP	EO	SE	HP	EO	SE	HP	EO
Tg [°C]	63.3	64.2	63.7	56.4	57.4	52.3	106.8	107.7	107.8
CTE 1 [µm/(m °C)] * T1@ [°C]	91.2 55.0	85.9 50.0	90.8 50.0	126.9 50.0	96.1 40.0	99.0 40.0	137.4 70.0	140.3 70.0	121.2 70.0
CTE 2 [µm/(m °C)] * T2@ [°C]	187.7 130.0	189.9 130.0	187.9 129.9	188.2 129.8	214.8 130.0	200.5 129.3	10,324 128.7	10,102 127.7	11,780 127.0

* T1@ and T2@ indicate the temperature at which CTE1 or CTE2 was evaluated.

**Table 4:** Glass transition temperature (Tg) and changes in Tg (ΔTg) obtained from DMA tan (delta) graphs of 3D-printed samples (PLA, PLA + FC, and PETG) sterilized by HP and EO.

Material	PLA			PLA + FC			PETG		
Treatment	Control	HP	EO	Control	HP	EO	Control	HP	EO
Tg (°C)	64.6	64.1	65.3	60.2	59.9	59.7	73.6	75.4	74.4
ΔTg (°C)		−0.5	0.7		−0.2	−0.5		1.7	0.7

**Table 5:** Thermal transitions and enthalpy values obtained from DSC analysis of 3D-printed PLA, PLA + CF, and PETG before and after sterilization by HP and EO.

Material	Treatment	Tg (°C)	Tm (°C)	ΔHcc (J·g^−1^)	ΔHm (J·g^−1^)	ΔHm^0^ (J·g^−1^)	Xc (%)
PLA	Control	64.3	164.7	17.1	32.5	93.0	16.6
	HP	64.8	163.8	11.2	44.8	93.0	36.1
	EO	63.1	164.7	13.5	34.2	93.0	22.3
PLA + CF	Control	60.6	169.2	13.8	21.3	79.1	9.5
	HP	61.7	169.5	9.9	21.7	79.1	14.9
	EO	61.1	169.1	5.8	19.0	79.1	16.7
PETG	Control	–	–	–	–	–	–
	HP	–	–	–	–	–	–
	EO	–	–	–	–	–	–

Note: (–) indicates no detectable transition within the analyzed temperature range, consistent with the amorphous nature of PETG.

## Data Availability

All data are available through e-mail (jmfuentes@uce.edu.ec).

## References

[1] Pérez Davila S., González Rodríguez L., Chiussi S., Serra J., González P. (2021). How to Sterilize Polylactic Acid Based Medical Devices?. Polymers.

[2] Oth O., Dauchot C., Orellana M., Glineur R. (2019). How to Sterilize 3D Printed Objects for Surgical Use? An Evaluation of the Volumetric Deformation of 3D-Printed Genioplasty Guide in PLA and PETG after Sterilization by Low-Temperature Hydrogen Peroxide Gas Plasma. Open Dent. J..

[3] Neijhoft J., Henrich D., Kammerer A., Janko M., Frank J., Marzi I. (2023). Sterilization of PLA after Fused Filament Fabrication 3D Printing: Evaluation on Inherent Sterility and the Impossibility of Autoclavation. Polymers.

[4] Zhang W., Lin X., Jiang J. (2022). Dimensional accuracy of 3D printing navigation templates of chemical-based sterilisation. Sci. Rep..

[5] Rynio P., Galant K., Wójcik Ł., Grygorcewicz B., Kazimierczak A., Falkowski A., Gutowski P., Dołęgowska B., Kawa M. (2022). Effects of Sterilization Methods on Different 3D Printable Materials for Templates of Physician-Modified Aortic Stent Grafts Used in Vascular Surgery—A Preliminary Study. Int. J. Mol. Sci..

[6] Fuentes J.M. (2024). Efectos de esterilización por peróxido de hidrógeno y óxido de etileno en materiales impresos en 3d: Revisión bibliográfica. Interciencia.

[7] Told R., Ujfalusi Z., Pentek A., Kerenyi M., Banfai K., Vizi A., Szabo P., Melegh S., Bovari-Biri J., Pongracz J.E. (2022). A state-of-the-art guide to the sterilization of thermoplastic polymers and resin materials used in the additive manufacturing of medical devices. Mater. Des..

[8] Savaris M., Santos V.D., Brandalise R.N. (2016). Influence of different sterilization processes on the properties of commercial poly(lactic acid). Mater. Sci. Eng. C.

[9] Valls-Esteve A., Lustig-Gainza P., Adell-Gomez N., Tejo-Otero A., Englí-Rueda M., Julian-Alvarez E., Navarro-Sureda O., Fenollosa-Artés F., Rubio-Palau J., Krauel L. (2023). A state-of-the-art guide about the effects of sterilization processes on 3D-printed materials for surgical planning and medical applications: A comparative study. Int. J. Bioprint..

[10] Shilov S.Y., Rozhkova Y.A., Markova L.N., Tashkinov M.A., Vindokurov I.V., Silberschmidt V.V. (2022). Biocompatibility of 3D-Printed PLA, PEEK and PETG: Adhesion of Bone Marrow and Peritoneal Lavage Cells. Polymers.

[11] Gradwohl M., Chai F., Payen J., Guerreschi P., Marchetti P., Blanchemain N. (2021). Effects of Two Melt Extrusion Based Additive Manufacturing Technologies and Common Sterilization Methods on the Properties of a Medical Grade PLGA Copolymer. Polymers.

[12] Karipidou N., Tzavellas A.-N., Petrou N., Katrilaka C., Theodorou K., Pitou M., Tsiridis E., Choli-Papadopoulou T., Aggeli A. (2023). Comparative studies of sterilization processes for sensitive medical nano-devices. Mater. Today Proc..

[13] Marturello D.M., Déjardin L.M. (2023). Post-sterilization Dimensional Accuracy of Methacrylate Monomer Biocompatible Three-Dimensionally Printed Mock Surgical Guides. Vet. Comp. Orthop. Traumatol..

[14] Popescu D., Baciu F., Vlăsceanu D., Cotruţ C.M., Marinescu R. (2020). Effects of multiple sterilizations and natural aging on the mechanical behavior of 3D-printed ABS. Mech. Mater..

[15] SUNLU|Affordable 3D Printing Filaments, Filament Dryer and 3D Resins, Affordable 3D Printing Filaments and Resins. https://www.sunlu.com/.

[16] La Solución CAD 3D para el Diseño y Desarrollo de Productos. https://www.solidworks.com/.

[17] (2012). Plastics—Determination of Tensile Properties Part 2: Test Conditions for Moulding and Extrusion Plastics.

[18] (2014). Sterilization of Health-Care Products—Ethylene Oxide—Requirements for the Development, Validation and Routine Control of a Sterilization Process for Medical Devices.

[19] (2022). Sterilization of Health Care Products—Low Temperature Vaporized Hydrogen Peroxide—Requirements for the Development, Validation and Routine Control of a Sterilization Process for Medical Devices.

[20] Shimadzu Corporation. https://www.shimadzu.com/.

[21] (2018). Standard Test Method for Determining the Charpy Impact Resistance of Notched Specimens of Plastics.

[22] Ibertest, Péndulo de Impacto Charpy Instrumentado—Ibertest. https://www.ibertest.es/products/pendulo-de-impacto-charpy-instrumentado/.

[23] TA Instruments Materials Science—TA Instruments. https://www.tainstruments.com/.

[24] Liu Y., Jiang S., Yan W., He M., Qin J., Qin S., Yu J. (2020). Crystallization Morphology Regulation on Enhancing Heat Resistance of Polylactic Acid. Polymers.

[25] Socrates G., Socrates G. (2001). Infrared and Raman Characteristic Group Frequencies: Tables and Charts.

[26] Origin: Data Analysis and Graphing Software. https://www.originlab.com/origin.

[27] Fuentes M., Cadena H., Flor O. DataSet Sterilization Processes (Hydrogen Peroxide/Ethylene Oxide) on Commercial 3D-Printed PLA, PLA-FC. Mendeley Data. https://data.mendeley.com/datasets/c9npfrpyn5/1.

[28] Pérez-Davila S., González-Rodríguez L., Lama R., López-Álvarez M., Oliveira A.L., Serra J., Novoa B., Figueras A., González P. (2022). 3D-Printed PLA Medical Devices: Physicochemical Changes and Biological Response after Sterilisation Treatments. Polymers.

[29] Velghe I., Buffel B., Vandeginste V., Thielemans W., Desplentere F. (2023). Review on the Degradation of Poly(lactic acid) during Melt Processing. Polymers.

[30] Bosc R., Tortolano L., Hersant B., Oudjhani M., Leplay C., Woerther P.L., Aguilar P., Leguen R., Meningaud J.-P. (2021). Bacteriological and mechanical impact of the Sterrad sterilization method on personalized 3D printed guides for mandibular reconstruction. Sci. Rep..

[31] Cao D. (2024). Increasing strength and ductility of extruded polylactic acid matrix composites using short polyester and continuous carbon fibers. Int. J. Adv. Manuf. Technol..

[32] Liu M., Liang T., Zhao Y., Yang C., Shi M., Wang S. (2025). A New Method of Graphene Oxide and Multi—Walled Carbon Nanotube Grafting for Improving Mechanical Properties and Thermal Stability of Carbon Fiber—Polylactic Acid Composites. Polym. Compos..

[33] Cressall S., Phillips C.O., Al-Shatty W., Deganello D. (2024). The effect of high-intensity gamma radiation on PETG and ASA polymer-based fused deposition modelled 3D printed parts. J. Mater. Sci..

[34] Khan A., Sapuan S.M., Zainudin E.S., Zuhri M.Y.M. (2024). Physical, mechanical and thermal properties of novel bamboo/kenaf fiber-reinforced polylactic acid (PLA) hybrid composites. Compos. Commun..

[35] Xing D., Wang H., Tao Y., Zhang J., Li P., Koubaa A. (2025). 3D—Printing continuous plant fiber/polylactic acid composites with lightweight and high strength. Polym. Compos..

[36] Tümer E.H., Erbil H.Y. (2021). Extrusion-Based 3D Printing Applications of PLA Composites: A Review. Coatings.

[37] Bouguermouh K., Habibi M., Laperrière L., Li Z., Abdin Y. (2024). 4D-printed PLA-PETG polymer blends: Comprehensive analysis of thermal, mechanical, and shape memory performances. J. Mater. Sci..

[38] Habiba R., Amaro A., Moura C., Silva R., Trindade D., Antão A., Martins R., Malça C., Branco R., Martins Amaro A., Roseiro L., Messias A.L., Gomes B., Almeida H., António Castro M., Neto M.A., De Fátima Paulino M., Maranha V. (2023). Impact Resistance of Additively Manufactured Polymeric Materials for Biomedical Applications. Proceedings of the 10th Congress of the Portuguese Society of Biomechanics.

[39] Niaza K.V., Senatov F.S., Stepashkin A., Anisimova N.Y., Kiselevsky M.V. (2017). Long-Term Creep and Impact Strength of Biocompatible 3D-Printed PLA-Based Scaffolds. Nano Hybrids Compos..

[40] Majka T.M., Leszczyńska A., Pielichowski K., Huang X., Zhi C. (2016). Thermal Stability and Degradation of Polymer Nanocomposites. Polymer Nanocomposites.

[41] Jansen J.C., Drioli E., Giorno L. (2015). Glass Transition Temperature (T g). Encyclopedia of Membranes.

